# Risk Factors for Peripheral Nerve Injury Following Revision Total Hip Arthroplasty in 112,310 Patients

**DOI:** 10.3390/jcm13061779

**Published:** 2024-03-20

**Authors:** Xiao T. Chen, Shane S. Korber, Kevin C. Liu, Brandon S. Gettleman, Shane Shahrestani, Nathanael D. Heckmann, Alexander B. Christ

**Affiliations:** 1 Department of Orthopaedic Surgery, Mayo Clinic, Rochester, MN 55905, USA; 2 Department of Orthopaedic Surgery, Keck School of Medicine of University of Southern California, Los Angeles, CA 90089, USA; 3 University of South Carolina School of Medicine, Columbia, SC 29209, USA; 4 Department of Orthopaedic Surgery, University of California at Los Angeles, Santa Monica, CA 90404, USA; 5 Department of Surgery, VA Greater Los Angeles Healthcare System, Los Angeles, CA 90073, USA

**Keywords:** revision total hip arthroplasty, peripheral nerve injury, epidemiology, national database

## Abstract

**Background**: Peripheral nerve injury (PNI) following revision total hip arthroplasty (rTHA) can be a devastating complication. This study assessed the frequency of and risk factors for postoperative PNI following rTHA. **Methods:** Patients who underwent rTHA from 2003 to 2015 were identified using the National Inpatient Sample (NIS). Demographics, medical history, surgical details, and complications were compared between patients who sustained a PNI and those who did not, to identify risk factors for the development of PNI after rTHA. **Results:** Overall, 112,310 patients who underwent rTHA were identified, 929 (0.83%) of whom sustained a PNI. Univariate analysis found that younger patients (*p* < 0.0001), females (*p* = 0.025), and those with a history of flexion contracture (0.65% vs. 0.22%, *p* = 0.005), hip dislocation (24.0% vs. 18.0%, *p* < 0.001), and spine conditions (4.8% vs. 2.7%, *p* < 0.001) had significantly higher rates of PNI. In-hospital complications associated with PNI included postoperative hematoma (2.6% vs. 1.2%, *p* < 0.0001), postoperative seroma (0.75% vs. 0.30%, *p* = 0.011), superficial wound dehiscence (0.65% vs. 0.23%, *p* = 0.008), and postoperative anemia (36.1% vs. 32.0%, *p* = 0.007). Multivariate analysis demonstrated that a history of pre-existing spine conditions (aOR: 1.7; 95%-CI: 1.3–2.4, *p* < 0.001), prior dislocation (aOR 1.5; 95%-CI: 1.3–1.7, *p* < 0.001), postoperative anemia (aOR 1.2; 95%-CI: 1.0–1.4, *p* = 0.01), and hematoma (aOR 2.1; 95%-CI: 1.4–3.2, *p* < 0.001) were associated with increased risk for PNI. **Conclusions:** Our findings align with the existing literature, affirming that sciatic nerve injury is the prevailing neuropathic complication after total hip arthroplasty (THA). Furthermore, we observed a 0.83% incidence of PNI following rTHA and identified pre-existing spine conditions, prior hip dislocation, postoperative anemia, or hematoma as risk factors. Orthopedic surgeons may use this information to guide their discussion of PNI following rTHA, especially in high-risk patients.

## 1. Introduction

Peripheral nerve injury (PNI) following total hip arthroplasty (THA) is a rare complication with potential for significant morbidity [1]. PNIs can occur during patient positioning or intraoperatively secondary to traction, ischemia, or transection [1,2,3]. While approximately one-third to one-half of patients with this complication achieve satisfactory functional recovery after their index surgery, the majority suffer from chronic pain and/or disability [4,5,6,7,8,9]. Despite advancements in surgical technique and instrumentation, the prevalence of PNI in the primary THA setting is estimated to be between 0.17 and 3.7%, though this rate has been reported as high as 7.6% in revision THA (rTHA) [10]. The sciatic and femoral nerves are the most commonly injured with incidences as high as 8% and 2.3%, respectively [11].

There are limited data regarding risk factors for PNI, with most data originating from small, retrospective cohort studies [1,12,13]. The largest study investigating risk factors for PNI was performed by Christ et al., who found a PNI rate of 0.23% in 207,000 primary THA patients and identified pre-existing spinal conditions, prior hip dislocation, and diabetes with chronic conditions as risk factors for the development of postoperative PNI [14]. Similarly, Shetty et al. performed a case-control study on 93 patients who developed PNI following THA and found age <45 years, tobacco use, history of spinal surgery or disease, and increased surgical time to be risk factors for PNI [15]. However, no study to date has attempted to characterize risk factors for PNI in rTHA only, which itself has been cited as a risk factor [16].

As rTHA volume is projected to increase dramatically over the next several decades [17], it is crucial to identify potential predictors of PNI to guide decision-making and improve patient outcomes. Therefore, the purpose of this study was to identify independent risk factors for PNI following rTHA using a large, nationally representative database.

## 2. Materials and Methods

### 2.1. National Inpatient Sample

The National Inpatient Sample (NIS) is a nationally representative database provided by the Healthcare Cost and Utilization Project (HCUP) and the Agency for Healthcare Research and Quality (AHRQ) that contains inpatient data from hospitals in 47 states. International Classification of Diseases, Ninth Revision (ICD-9) and International Classification of Diseases, Tenth Revision (ICD-10) codes represent over 100 data elements including patient demographics, inpatient complications, and discharge data. This study was exempt from institutional review board approval as all patient data within the NIS are de-identified.

### 2.2. Identification of Study Cohorts

A retrospective cohort study was conducted on all patients within the 2003–2015 NIS who underwent rTHA, identified using the following ICD-9 procedure codes: 0070, 0071, 0072, 0073, and 8153. Exclusion criteria were patients who underwent a primary THA or hemiarthroplasty and oncology patients who underwent THA for osseous pelvic neoplasms (ICD-9 procedure codes: 1706, 1707, 1709, 8151, 8152) (Supplemental Table S1). The rTHA cohort was subsequently divided into two groups: those who developed a PNI and those who did not. To identify patients with PNI following rTHA, the following ICD-9 Diagnosis codes were used: sciatic nerve injury (956.0); femoral nerve injury (956.1); posterior tibial nerve injury (956.2); peroneal nerve injury (956.3); cutaneous sensory nerve, lower limb (956.4); other unspecified nerve(s) of pelvic girdle and lower limb (956.8); unspecified nerve of pelvic girdle and lower limb (956.9); lesion of the sciatic nerve (355.0); meralgia paresthetica (355.1); other lesion of femoral nerve (355.2); lesion of lateral popliteal nerve (355.3); lesion of medial popliteal nerve (355.4); other mononeuritis of lower limb (355.7); or mononeuritis of lower limb, unspecified (355.8) (Supplemental Table S2).

### 2.3. Data Collection

Patient demographics (age, sex, race, surgical indication), comorbidities, pertinent medical history, and in-hospital complications were assessed and compared between the two cohorts (Supplemental Table S3).

### 2.4. Data Analyses

Univariate analysis was performed to assess differences between patients who sustained a PNI following revision THA versus those who did not. Categorical variables were presented as frequencies and percentages and compared using Chi-square tests or Fisher’s exact tests. Univariate logistic regression was performed to identify potential risk factors for the development of PNI. Factors approaching a significant difference between cohorts (*p* < 0.100) were then fitted into a multivariable logistic regression to identify independent risk factors for the development of PNI after rTHA and adjust for potential confounding factors. The results of this multivariate analysis were presented as odds ratios (OR) and 95% confidence intervals. All tests were two-sided and *p*-values < 0.05 were considered statistically significant. Statistical analyses were performed using STATA (version 16.1; StataCorp, College Station, TX, USA).

## 3. Results

A total of 112,310 rTHAs were identified and 929 (0.83%) patients had a new PNI following rTHA. Of those diagnosed with a PNI, there were 284 (30.6%) sciatic nerve, 139 (15.0%) popliteal nerve, 58 (6.2%) femoral nerve, 19 (2.1%) peroneal nerve, and 17 (1.8%) unspecified lower limb nerve injuries. Additionally, 416 (44.8%) PNI patients were diagnosed with mononeuritis of the lower limb.

On univariate analysis, PNI was more common in patients who were younger (*p* < 0.0001) and female (*p* = 0.025) (Table 1). There was no association of patient race with PNI (*p* = 0.07). Furthermore, patients who sustained a postoperative PNI were more likely to have a history of flexion contracture (0.65% vs. 0.22%, *p* = 0.005), prior hip dislocation (24.0% vs. 18.0%, *p* < 0.0001), or spine condition (4.8% vs. 2.7%, *p* < 0.0001) than those who did not develop a PNI. Patients who developed a PNI were more likely to have concomitant peripheral vascular disease (4.3% vs. 2.9%, *p* = 0.011), obesity (14.1% vs. 11.1%, *p* = 0.004), depression (17.2% vs. 13.7%, *p* = 0.002), drug abuse (2.1% vs. 1.3%, *p* = 0.031), or metastatic cancer (1.2% vs. 0.5%, *p* = 0.002) (Table 2).

In-hospital complications associated with an increased rate of PNI included postoperative hematoma (2.6% vs. 1.2%, *p* < 0.0001), postoperative seroma (0.75% vs. 0.30%, *p* = 0.011), superficial wound dehiscence (0.65% vs. 0.23%, *p* = 0.008), and postoperative anemia (36.1% vs. 32.0%, *p* = 0.007). Those who did not sustain a postoperative PNI had significantly increased rates of respiratory failure (1.3% vs. 3.2%, *p* = 0.001) and acute renal failure (2.8% vs. 4.1%, *p* = 0.045) compared to the postoperative PNI cohort (Table 2).

After accounting for confounders on multivariate analysis, younger age was identified as a risk factor for PNI after rTHA. Compared to patients under 50, those aged 50–59 (adjusted odds ratio [aOR]: 0.75, 95%-Confidence interval [95%-CI]: 0.60–0.95, *p* = 0.02), 70–79 (aOR 0.71; 95%-CI: 0.57–0.89, *p* = 0.003), and over 80 (aOR 0.56; 95%-CI: 0.43–0.72, *p* < 0.001) were progressively less likely to have a PNI following rTHA. Furthermore, a history of pre-existing spine conditions (aOR: 1.74; 95%-CI: 1.29–2.36, *p* < 0.001) or hip dislocation (aOR 1.48; 95%-CI: 1.26–1.74, *p* < 0.001) were associated with increased risk for PNI. In-hospital complications associated with increased risk of PNI following rTHA included postoperative anemia (aOR 1.20; 95%-CI: 1.04–1.38, *p* = 0.01) and hematoma (aOR 2.12; 95%-CI: 1.40–3.22, *p* < 0.001). Postoperative seroma trended towards significance as a positive predictor of PNI (aOR 2.01; 95%-CI: 0.98–4.47, *p* = 0.057). Once confounders were accounted for, sex and medical comorbidities were no longer found to be significant risk factors for PNI (Table 3).

## 4. Discussion

This study assessed the incidence of PNI following rTHA in a nationally representative sample of over 112,000 cases. The incidence of immediately diagnosed PNI in this cohort was 0.83%, which is notably higher than the 0.23% rate in primary THAs in a large database study by Christ et al. [14]. This rate was consistent with the 0–7.6% rate of PNI in rTHA reported by older studies [11,18], especially the reported incidence of 1.4–3.2% in higher-powered prior studies that included 250–700 rTHAs [12,19,20]. The reason for the lower incidence of PNI in this contemporary cohort of rTHA patients is unclear and warrants further investigation. Regardless, this information is useful for counseling rTHA patients that PNI following surgery is unlikely but increased compared to their index surgery.

A major finding of this study following multivariate analysis was an increased risk of PNI in rTHA patients with pre-existing spinal conditions (OR = 1.7). The previous literature has reported similarly increased risks of PNI in primary THA patients with prior spinal pathology [14], with the thought that nerves with pre-existing spinal compression may become less tolerant to a second compressive insult, dubbed the “double crush syndrome” [11]. Giving credence to this phenomenon, Chughtai et al. reported improved functional outcomes in patients diagnosed with PNI following THA who were treated with nerve decompression surgery [21]. While limited current data suggest that rTHA patients who develop PNI could be appropriately treated with nerve decompression, there is no evidence on whether preoperative prophylactic decompression in rTHA for elective indications may potentially improve outcomes and decrease the risk of PNI. Furthermore, to the best of the author’s knowledge, there are no data on the utility of undergoing elective decompressive spine surgery before rTHA to decrease proximal nerve stenosis. There are a considerable number of unknown variables concerning perioperative surgical options that warrant further investigation.

A history of dislocation was also found to be a risk factor for PNI following rTHA following multivariate analysis, which may be explained by stretch, traction, and/or direct trauma affecting nerves during dislocation events. Additional studies have reported postoperative PNI as a result of over-lengthening in patients treated for hip dislocations [22]. However, these proposed mechanisms are not well established in the literature, and a history of dislocation is unlikely to be the underlying etiology of postoperative PNI. Rather, prior dislocation may alter surgical anatomy or soft tissue tension, leading to a higher risk of intraoperative nerve injury [2,13]. It is also possible that prior dislocations may have caused preoperative PNIs that were documented postoperatively and thereby coded as a complication following the revision surgery. Further investigation is needed to clarify the association between prior dislocation and PNI. This finding emphasizes the importance of appropriate implant selection, sizing, and placement at each patient’s index THA to avoid dislocation and associated mechanical complications.

Previous large-scale studies have found a strong association between younger age (<50 years) and development of PNI after THA [1,23]. Younger patients are more likely to have greater musculature and body mass, which increases both mean operative time and the retraction force required to obtain adequate surgical exposure. Younger patients may also be presenting for surgery earlier due to more severe disease, which could independently increase the chance of postoperative PNI [15]. In this study, nearly every increased decade of age was associated with a concomitant decrease in the odds of developing a PNI after rTHA. These results are consistent with those reported by Christ et al. [14], who performed the same analysis on primary THA patients. Preoperative counseling of younger patients—especially those with larger body habitus or more severe disease—should include a discussion on their elevated risk of developing PNI after both primary and rTHA.

This study identified two in-hospital complications following multivariate analysis associated with PNI after rTHA: postoperative anemia and hematoma. Postoperative seroma trended towards significance as well, but the analysis was underpowered due to the rarity of this complication. Seroma and hematoma likely act by the same mechanism, creating a compressive effect on adjacent peripheral nerves, leading to injury. Prior studies have found an increased risk of PNI in patients on stronger anticoagulation [15], and it is no surprise that hematomas around the sciatic nerve have been thought to contribute to PNI [24]. Anemia secondary to blood loss may also be associated with hypoxia and ischemia around nerves, further sensitizing them and decreasing their threshold for injury. These findings stress the importance of efficient surgical technique, adequate hemostasis before closure, and the use of all possible modalities to minimize blood loss.

There is some debate within the literature on whether female sex is an independent risk factor for PNI. A few studies have found female gender to be associated with PNI following THA [11,23] whereas larger, more recent studies have found no such association [1,25]. The database study of over 207,000 primary THAs by Christ et al. also found no difference in PNI between genders [14]. In this study, female sex was initially associated with greater rates of PNI following rTHA on univariate analysis but was no longer a significant risk factor after accounting for confounders on multivariate analysis. The association between sex and PNI after THA is likely spurious, at best, and there are likely multiple confounding factors contributing to the conflicting findings reported in the literature.

The surgical approach is a variable that was not assessed in this study but remains highly relevant in the discussion of PNI following rTHA. Notably, multiple prior studies have reported a higher incidence of PNI following usage of the posterior approach compared to the anterior approach [1,25]. On the other hand, the anterior approach has been associated with a greater risk of lateral femoral cutaneous nerve dysfunction [26,27]. Furthermore, there is considerable debate about the risk of periprosthetic hip dislocation with different surgical approaches. Historically, the anterior approach has been associated with superior stability and lower dislocation rates compared to the posterior approach [28], though recent studies suggest that these differences may not be clinically or statistically meaningful [29,30,31,32]. As previously discussed, the history of hip dislocation itself appears to be a risk factor for PNI, so the surgical approach may be a confounding factor. Regardless, the risk of PNI should not dictate the approach an orthopedic surgeon takes on performing their primary or rTHA. Rather, as contemporary patients inquire more frequently about their orthopedic surgeon’s preferred surgical approach, the risk of PNI with each approach should be discussed.

This study has several limitations. As with any retrospective database study, the data in this study are limited by the accuracy of medical coding. Additionally, while this study is high-powered, it lacks granularity on patient variables such as the number of prior rTHAs, body mass index, or degree of spinal pathology, which would have allowed for stratification of the data for further sub-analyses. Furthermore, the NIS does not contain patient-reported outcomes such as pain and function or have variables such as surgical approach, intraoperative blood loss, or operative time, which limits the ability to make clinical conclusions. There was also no information on the specific revision implants used or radiographic data to assess variables such as implant positioning. This study also only includes data from 2003 to 2015, and future studies may find different results as surgical techniques and technologies continue to improve. However, the change to ICD-10 coding in late 2015 made identifying rTHAs extremely inconsistent, so this study only investigated patients with rTHA as defined by a validated set of ICD-9 codes. Finally, data within the study are limited to short-term inpatient stays, so while acute postoperative PNIs were appropriately queried, there are no data on long-term recovery or outcomes.

## 5. Conclusions

In conclusion, this is one of the largest database studies to date that provides a high-power analysis assessing the incidence and risk factors for PNI following rTHA. Our findings align with the existing literature, affirming that sciatic nerve injury is the prevailing neuropathic complication after total hip arthroplasty (THA). Furthermore, we observed that pre-existing spine conditions, a history of hip dislocation, and in-hospital complications such as postoperative anemia and hematoma were associated with developing postoperative PNI. These data provide valuable information to orthopedic surgeons for identifying and counseling higher-risk patients and may help guide potential preoperative optimization.

## Figures and Tables

**Table 1:** Demographic comparisons between those who had a postoperative PNI and those who did not.

	PNI (−) (n = 111,381)		PNI (+) (n = 929)		
	Mean or n	SD or %	Mean or n	SD or %	*p*-Value
Patient age at procedure					
<50	11,862	10.65%	126	13.56%	<0.001
50–59	20,126	18.07%	167	17.98%	
60–69	27,130	24.36%	252	27.13%	
70–79	29,811	26.76%	240	25.83%	
80+	22,452	20.16%	144	15.50%	
Patient gender					
Female	64,060	57.51%	569	61.25%	0.025
Male	47,160	42.34%	360	38.75%	
Race					
White	79,720	71.57%	684	73.63%	0.07
Black	6622	5.95%	43	4.63%	
Hispanic	3373	3.03%	38	4.09%	
Asian or Pacific Islander	775	0.70%	omitted	0.32%	
Native American	358	0.32%	omitted	0.43%	
Other	1730	1.55%	20	2.15%	
Surgical indication					
Fracture	3915	3.51%	30	3.23%	0.001
Septic failure	7513	6.75%	39	4.20%	
Aseptic failure	74,306	66.71%	625	67.28%	
Other failure	11,792	10.59%	128	13.78%	

**Table 2:** Comorbidity comparison between those who had a postoperative PNI and those who did not.

	PNI (−) (n = 111,381)		PNI (+) (n = 929)		
	Mean or n	SD or %	Mean or n	SD or %	*p*-Value
Elixhauser Comorbidity Index					
Congestive heart failure	6280	5.66%	45	4.87%	0.301
Cardiac arrhythmias	4695	4.22%	31	3.34%	0.184
Valvular disease	5405	4.87%	39	4.22%	0.36
Pulmonary circulation disorders	1554	1.40%	Omitted	0.76%	0.097
Peripheral vascular disorders	3232	2.91%	40	4.33%	0.011
Hypertension (combined)	65,381	58.92%	525	56.82%	0.195
Paralysis	894	0.81%	6	0.65%	0.596
Other neurological disorders	6741	6.08%	54	5.84%	0.77
Chronic pulmonary disease	18,351	16.54%	145	15.69%	0.491
Diabetes, uncomplicated	15,400	13.88%	139	15.04%	0.308
Diabetes, complicated	1805	1.63%	Omitted	0.97%	0.118
Hypothyroidism	15,743	14.19%	144	15.58%	0.226
Renal failure	6466	5.83%	48	5.19%	0.414
Liver disease	1875	1.69%	14	1.52%	0.682
Peptic ulcer disease excluding bleeding	29	0.03%	Omitted	0.00%	0.623
AIDS	174	0.16%	Omitted	0.11%	0.71
Lymphoma	529	0.48%	Omitted	0.54%	0.777
Metastatic cancer	524	0.47%	11	1.19%	0.002
Solid tumor without metastasis	730	0.66%	Omitted	0.32%	0.211
Rheumatoid arthritis/collagen vascular diseases	7652	6.90%	71	7.68%	0.347
Coagulopathy	3932	3.54%	34	3.68%	0.824
Obesity	12,310	11.09%	130	14.07%	0.004
Weight loss	2261	2.04%	15	1.62%	0.374
Fluid and electrolyte disorders	15,074	13.58%	786	85.06%	0.233
Blood loss anemia	2442	2.20%	24	2.60%	0.413
Deficiency anemias	18,160	16.37%	163	17.64%	0.297
Alcohol abuse	2302	2.07%	17	1.84%	0.618
Drug abuse	1398	1.26%	19	2.06%	0.031
Psychoses	3121	2.81%	23	2.49%	0.553
Depression	15,150	13.65%	159	17.21%	0.002
In-hospital complication					
Periprosthetic fracture	6039	5.42%	41	4.41%	0.176
Periprosthetic joint infection	11,506	10.33%	79	8.50%	0.068
Postprocedural hemorrhage	1484	1.33%	15	1.61%	0.455
Postprocedural hematoma	1318	1.18%	24	2.58%	<0.0001
Postprocedural seroma	329	0.30%	Omitted	0.75%	0.011
Wound dehiscence, superficial	254	0.23%	Omitted	0.65%	0.008
Wound dehiscence, deep	367	0.33%	Omitted	0.22%	0.545
Pressure ulcer	1903	1.71%	17	1.83%	0.776
Acute myocardial infarction	678	0.61%	Omitted	0.54%	0.783
Pneumonia	1406	1.26%	Omitted	0.75%	0.166
Respiratory failure	3529	3.17%	12	1.29%	0.001
Acute renal failure	4575	4.11%	26	2.80%	0.045
Cerebral infarction	244	0.22%	Omitted	0.11%	0.469
Inpatient death	671	0.60%	Omitted	0.43%	0.499
Postoperative ileus	1115	1.00%	Omitted	0.97%	0.922
Urinary tract infection	6473	5.81%	49	5.27%	0.486
Postoperative Anemia	35,584	31.95%	335	36.06%	0.007
Past medical history of					
Flexion contracture	241	0.22%	Omitted	0.65%	0.005
Valgus	1	0.00%	Omitted	0.00%	0.927
Varus	6	0.01%	Omitted	0.00%	0.823
Hip dislocation	20,064	18.01%	223	24.00%	<0.0001
Avascular Necrosis	1428	1.28%	13	1.40%	0.752
Juvenile Idiopathic Arthritis	385	0.35%	Omitted	0.54%	0.32
Rheumatoid Arthritis	5204	4.67%	50	5.38%	0.308
Systemic Lupus Erythematosus	1169	1.05%	Omitted	0.54%	0.126
End Stage Renal Disease	646	0.58%	Omitted	0.65%	0.792
Hardware removal	88	0.08%	Omitted	0.22%	0.144
Mechanical complications	40,542	36.40%	336	36.17%	0.884
Spondyloarthropathy	580	0.52%	Omitted	0.65%	0.598
Spine condition	2974	2.67%	45	4.84%	<0.0001

**Table 3:** Logistic regression model for risk factors for PNI.

	Adjusted Odds Ratio	95%-CI	*p*-Value
Age category			
50–59 vs. <50	0.75	0.60–0.95	0.018
60–69 vs. <50	0.83	0.67–1.03	0.097
70–79 vs. <50	0.71	0.57–0.89	0.003
80+ vs. <50	0.56	0.43–0.72	<0.001
Female vs. Male	1.14	1.00–1.31	0.057
Elixhauser Comorbidity Index			
Congestive heart failure	0.94	0.69–1.28	0.684
Chronic pulmonary disease	0.88	0.74–1.06	0.168
Hypothyroidism	1.09	0.90–1.30	0.383
Liver disease	0.80	0.47–1.37	0.425
Weight loss	0.73	0.43–1.22	0.231
Fluid and electrolyte disorders	0.11	0.92–1.34	0.29
In-hospital complication			
Mechanical complications	1.10	0.95–1.27	0.193
Bleeding	1.14	0.68–1.91	0.626
Periprosthetic fracture	0.87	0.63–1.19	0.376
Postoperative anemia	1.20	1.04–1.38	0.01
Hematoma	2.12	1.40–3.22	<0.001
Seroma	2.09	0.98–4.47	0.057
Spine condition + valgus (1, yes; 0, no)			
0 + 1 vs. 0 + 0	-	-	-
1 + 0 vs. 0 + 0	1.74	1.29–2.36	<0.001
1 + 1 vs. 0 + 0	-	-	-
History of hip dislocation	1.48	1.26–1.74	<0.001
Rheumatoid arthritis	1.03	0.80–1.31	0.832

## Data Availability

Data from this study are available by purchasing and accessing the National Inpatient Sample. https://cdors.ahrq.gov/ (accessed on 26 September 2022).

## Supplementary Materials

The following supporting information can be downloaded at: https://www.mdpi.com/article/10.3390/jcm13061779/s1, Table S1: Inclusion and Exclusion Codes; Table S2: Peripheral Nerve Injury Codes; Table S3: Codes for Comorbidites, Prior Surgical History, and Complications.
