# Supplementary material for: Risk Factors for Peripheral Nerve Injury Following Revision Total Hip Arthroplasty in 112,310 Patients
Source: J Clin Med. 2024 Mar 20;13(6):1779. doi: 10.3390/jcm13061779 (PMC10971509; doi:10.3390/jcm13061779)
Supplement: Supplementary file 1 [file jcm-13-01779-s001.zip › jcm-2884758-supplementary.pdf]

Supplemental Table S1. Inclusion and Exclusion Codes

**Procedure**	
Revision total hip arthroplasty	"0070", "0071", "0072", "0073", "8153"
**Surgical indication**	
**Acute fracture**	
Fracture of neck of femur	"82000", "82001", "82002", "82003", "82009", "82010", "82011", "82012", "82013", "82019", "82020", "82021", "82022", "82030", "82031", "82032", "8208", "8209"
Fracture of other and unspecified parts of femur	"82100", "82101", "82110", "82111", "82120", "82121", "82122", "82123", "82129", "82130", "82131", "82132", "82133", "82139"
Fracture of patella	"8220", "8221"
Fracture of tibia and fibula	"82300", "82301", "82302", "82310", "82311", "82312", "82320", "82321", "82322", "82330", "82331", "82332", "82340", "82341", "82342", "82380", "82301", "82382",

	“82390”, “82391”, “82392”
Other multiple and ill-defined fractures of lower limb	“8270”, “8271”
Pathologic fracture of femur	“73314”, “73315”
Malunion and nonunion of fracture	“73381”, “73382”
Other and unspecified disorders of bone and cartilage	“73390”, “73391”, “73392”, “73393”, “73395”, “73396”, “73397”, “73398”, “73399”
**Septic failure**	
Infection and inflammatory reaction due to unspecified device, implant and graft	“99660”
Infection and inflammatory reaction due to internal joint prosthesis	“99666”
Infection and inflammatory reaction due to other internal orthopedic device, implant and graft	“99667”
Infection and inflammatory reaction due to other internal prosthetic device, implant, and graft	“99667”
Infected postoperative seroma	“99851”

Other postoperative infection	"99859"
**Aseptic failure**	
Other complications of procedures, not elsewhere classified	"99800", "99801", "99802", "99809", "99811", "99812", "99813", "9982", "99830", "99831", "99832", "99833", "9984", "99851", "99859", "9986", "9987", "99881", "99882", "99883", "99889", "9989"
Complications of medical care, not elsewhere classified	"9990", "9991", "9992", "99931", "99932", "99933", "99934", "99939", "99941", "99942", "99949", "99951", "99952", "99959", "99960", "99961", "99962", "99963", "99969", "99970", "99971", "99972", "99973", "99974", "99975", "99976", "99977", "99978", "99979", "99980", "99981", "99982", "99983", "99984", "99985", "99988", "99989", "9999"
Mechanical complication of internal orthopedic device, implant, and graft	"99640", "99641", "99642", "99643", "99644", "99645", "99646", "99647", "99649"
Mechanical complication due to other implant and internal	"99659"

device, not elsewhere classified	
Other symptoms referable to and specified disorders of joint, pelvic region and thigh	"71965", "71985", "71995"
Other derangement of joint	"71845"
Ankylosis of joint	"71855"
Unspecified intrapelvic protrusion of acetabulum	"71865"
Other complications of internal prosthetic device, implant, and graft	"99670", "99677", "99678", "99679"
**Other**	
Osteoarthrosis, unspecified whether generalized or localized, pelvic region and thigh	"71595"
Osteoarthrosis, localized, not specified whether primary or secondary, pelvic region and thigh	"71535"
Other acquired deformities of hip	"73630", "73631", "73632", "73639", "73681", "73689", "7369"
	Exclusion Diagnoses
Malignancy	"1706", "1707", "1709"

Primary total or partial hip arthroplasty	“8151”, “8152”

Supplemental Table S2. Peripheral Nerve Injury Codes

Nerve	ICD-9
Sciatic nerve	"9560"
Femoral nerve	"9561"
Posterior tibial nerve	"9562"
Peroneal nerve	"9563"
Cutaneous sensory nerve, lower limb	"9564"
Other unspecified nerve(s) of pelvic girdle and lower limb	"9568"
Unspecified nerve of pelvic girdle and lower limb	"9569"
Lesion of sciatic nerve	"3550"
Meralgia paresthetica	"3551"
Other lesion of femoral nerve	"3552"
Lesion of lateral popliteal nerve	"3553"
Lesion of medial popliteal nerve	"3554"
Causalgia of lower limb	"35571"
Other mononeuritis of lower limb	"3557"

Supplemental Table S3. Codes for Comorbidites, Prior Surgical History, and Complications

**Elixhauser comorbidity index**	
Congestive heart failure	"39891", "40211", "40291", "40411", "40413", "40491", "40493", "4280", "4281", "42820", "42821", "42822", "42823", "42830", "42831", "42832", "42833", "42840", "42841", "42842", "42843", "4289"
Cardiac arrhythmias	"42610", "42611", "42613", "4262", "4263", "4264", "42650", "42651", "42652", "42653", "4266", "4267", "42681", "42682", "42689", "4270", "4272", "42731", "42760", "4279", "7850", "V450", "V533"
Valvular disease	"09320", "09321", "09322", "09323", "09324", "3940", "3941", "3942", "3949", "3950", "3951", "3952", "3959", "3960", "3961", "3962", "3963", "3968", "3969", "3970", "3971", "4240", "4241", "4242", "4243", "42490", "42491", "42499", "7463", "7464", "7465", "7466", "V422", "V433"
Pulmonary circulation disorders	"4160", "4161", "4162", "4168", "4169", "4179"
Peripheral vascular disorders	"4400", "4401", "44020", "44021", "44022", "44023", "44024", "44029", "44030", "44031", "44032", "4404", "4408", "4409", "4412", "4414", "4417", "4419", "4430", "4431", "44321", "44322", "44323", "44324", "44329", "44381", "44382", "44389", "4439", "4471", "5571", "5579", "V434"

Hypertension (combined)	“4011”, “4019”, “40210”, “40290”, “40410”, “40490”, “40511”, “40519”, “40591”, “40599”
Paralysis	“34200”, “34201”, “34202”, “34210”, “34211”, “34212”, “34290”, “34291”, “34292”, “3430”, “3431”, “3432”, “3433”, “3434”, “3438”, “3439”, “34400”, “34401”, “34402”, “34403”, “34404”, “34409”, “3441”, “3442”, “34430”, “34431”, “34432”, “34440”, “34441”, “34442”, “3445”, “34460”, “34461”, “34481”, “34489”, “3449”
Other neurological disorders	“3319”, “3320”, “3334”,“ 3335”, “3340”, “3341”, “3342”, “3343”, “3344”, “3348”, “3349”, “3350”, “33510”, “33511”, “33519”, “33520”, “33521”, “33522”, “33523”, “33524”, “33529”, “3358”, “3359”, “340”, “3410”, “3411”, “34120”, “34121”, “34122”, “3418”, “3419”, “34500”, “34501”, “34510”, “34511”, “3452”, “3453”, “34540”, “34541”, “34550”, “34551”, “34580”, “34581”, “34590”, “34591”, “3481”, “3483”, “7803”, “7843”
Chronic pulmonary disease	“490”, “4910”, “49120”, “49121”, “49122”, “4918”, “4919”, “4920”, “4928”, “49300”, “49301”, “49302”, “49310”, “49311”, “49312”, “49320”, “49321”, “49322”, “49381”, “49382”, “49390”, “49391”, “4940”, “4941”, “4950”, “4951”, “4952”, “4953”, “4954”, “4955”, “4956”, “4957”, “4958”, “4959”, “496”, “500”, “501”, “502”, “503”, “504”, “505”, “5064”
Diabetes, uncomplicated	“25000”, “25001”, “25002”, “25003”, “25010”, “25011”, “25012”, “25013”, “25020”,

	"25021", "25022", "25023", "25030", "25031", "25032", "25033"
Diabetes, complicated	"25040", "25041", "25042", "25043", "25050", "25051", "25052", "25053", "25060", "25061", "25062", "25063", "25070", "25071", "25072", "25073", "25090", "25091", "25092", "25093"
Hypothyroidism	"243", "2440", "2441", "2442", "2448", "2449"
Renal failure	"40311", "40391", "40412", "40492", "585", "586", "V420", "V451", "V560", "V568"
Liver disease	"07032", "07033", "07054", "4560", "4561", "45620", "45621", "5710", "5712", "5713", "57140", "57141", "57142", "57149", "5715", "5716", "5718", "5719", "5723", "5728", "V427"
Peptic ulcer disease excluding bleeding	"53170", "53190", "53270", "53290", "53370", "53390", "53470", "53490", "V1271"
AIDS	"042"
Lymphoma	"20000", "20001", "20002", "20003", "20004", "20005", "20006", "20007", "20008", "20010", "20011", "20012", "20013", "20014", "20015", "20016", "20017", "20018", "20020", "20021", "20022", "20023", "20024", "20025", "20026", "20027", "20028", "20030", "20031", "20032", "20033", "20034", "20035", "20036", "20037", "20038", "20040", "20041", "20042", "20043", "20044", "20045", "20046", "20047", "20048", "20050", "20051", "20052", "20053", "20054", "20055", "20056", "20057", "20058", "20060", "20061", "20062",

“20063”, “20064”, “20065”,
“20066”, “20067”, “20068”,
“20070”, “20071”, “20072”,
“20073”, “20074”, “20075”,
“20076”, “20077”, “20078”,
“20080”, “20081”, “20082”,
“20083”, “20084”, “20085”,
“20086”, “20087”, “20088”,
“20100”, “20101”, “20102”,
“20103”, “20104”, “20105”,
“20106”, “20107”, “20108”,
“20110”, “20111”, “20112”,
“20113”, “20114”, “20115”,
“20116”, “20117”, “20118”,
“20120”, “20121”, “20122”,
“20123”, “20124”, “20125”,
“20126”, “20127”, “20128”,
“20140”, “20141”, “20142”,
“20143”, “20144”, “20145”,
“20146”, “20147”, “20148”,
“20150”, “20151”, “20152”,
“20153”, “20154”, “20155”,
“20156”, “20157”, “20158”,
“20160”, “20161”, “20162”,
“20163”, “20164”, “20165”,
“20166”, “20167”, “20168”,
“20170”, “20171”, “20172”,
“20173”, “20174”, “20175”,
“20176”, “20177”, “20178”,
“20190”, “20191”, “20192”,
“20193”, “20194”, “20195”,
“20196”, “20197”, “20198”,
“20200”, “20201”, “20202”,
“20203”, “20204”, “20205”,
“20206”, “20207”, “20208”,
“20210”, “20211”, “20212”,
“20213”, “20214”, “20215”,
“20216”, “20217”, “20218”,
“20220”, “20221”, “20222”,
“20223”, “20224”, “20225”,
“20226”, “20227”, “20228”,
“20230”, “20231”, “20232”,
“20233”, “20234”, “20235”,
“20236”, “20237”, “20238”,
“20250”, “20251”, “20252”,
“20253”, “20254”, “20255”,
“20256”, “20257”, “20258”,
“20260”, “20261”, “20262”,
“20263”, “20264”, “20265”,
“20266”, “20267”, “20268”,
“20270”, “20271”, “20272”,
“20273”, “20274”, “20275”,
“20276”, “20277”, “20278”,
“20290”, “20291”, “20292”,
“20293”, “20294”, “20295”,

	"20296", "20297", "20298", "20300", "20301", "20380", "20381", "2386", "2733", "V1071", "V1072", "V1079"
Metastatic cancer	"1960", "1961", "1962", "1963", "1965", "1966", "1968", "1969", "1970", "1971", "1972", "1973", "1974", "1975", "1976", "1977", "1978", "1981", "1982", "1983", "1984", "1985", "1986", "1987", "19881", "19882", "19889", "1990", "1991"
Solid tumor without metastasis	"1400", "1401", "1403", "1404", "1405", "1406", "1408", "1409", "1410", "1411", "1412", "1413", "1414", "1415", "1416", "1418", "1419", "1420", "1421", "1422", "1428", "1429", "1430", "1431", "1438", "1439", "1440", "1441", "1448", "1449", "1450", "1451", "1452", "1453", "1454", "1455", "1456", "1458", "1459", "1460", "1461", "1462", "1463", "1464", "1465", "1466", "1467", "1468", "1469", "1470", "1471", "1472", "1473", "1478", "1479", "1480", "1481", "1482", "1483", "1488", "1489", "1490", "1491", "1498", "1499", "1500", "1501", "1502", "1503", "1504", "1505", "1508", "1509", "1510", "1511", "1512", "1513", "1514", "1515", "1516", "1518", "1519", "1520", "1521", "1522", "1523", "1528", "1529", "1530", "1531", "1532", "1533", "1534", "1535", "1536", "1537", "1538", "1539", "1540", "1541", "1542", "1543", "1548", "1550", "1551", "1552", "1560", "1561", "1562", "1568", "1569", "1570", "1571",

“1572”, “1573”, “1574”,
“1578”, “1579”, “1580”,
“1588”, “1589”, “1590”,
“1591”, “1598”, “1599”,
“1600”, “1601”, “1602”,
“1603”, “1604”, “1605”,
“1608”, “1609”, “1610”,
“1611”, “1612”, “1613”,
“1618”, “1619”, “1620”,
“1622”, “1623”, “1624”,
“1625”, “1628”, “1629”,
“1630”, “1631”, “1638”,
“1639”, “1640”, “1641”,
“1642”, “1643”, “1648”,
“1649”, “1650”, “1658”,
“1659”, “1700”, “1701”,
“1702”, “1703”, “1704”,
“1705”, “1706”, “1707”,
“1708”, “1709”, “1710”,
“1712”, “1713”, “1714”,
“1715”, “1716”, “1717”,
“1718”, “1719”, “1720”,
“1721”, “1722”, “1723”,
“1724”, “1725”, “1726”,
“1727”, “1728”, “1729”,
“1740”, “1741”, “1742”,
“1743”, “1744”, “1745”,
“1746”, “1748”, “1749”,
“1750”, “1759”, “179”, “1800”,
“1801”, “1808”, “1809”, “181”,
“1820”, “1821”, “1828”,
“1830”, “1832”, “1834”,
“1835”, “1838”, “1839”,
“1840”, “1841”, “1842”,
“1843”,“1844”, “1848”,
“1849”, “185”, “1860”,
“1869”, “1870”, “1871”,
“1872”, “1873”, “1874”,
“1875”, “1876”, “1877”,
“1878”, “1879”, “1880”,
“1881”, “1882”, “1883”,
“1884”, “1885”, “1886”,
“1887”, “1888”, “1889”,
“1890”, “1891”, “1892”,
“1893”, “1894”, “1898”,
“1899”, “1900”, “1901”,
“1902”, “1903”, “1904”,
“1905”, “1906”, “1907”,
“1908”, “1909”, “1910”,
“1911”, “1912”, “1913”,
“1914”, “1915”, “1916”,
“1917”, “1918”, “1919”,
“1920”, “1921”, “1922”,
“1923”, “1928”, “1929”,
“193”, “1940”, “1941”,

	"1943", "1944", "1945", "1946", "1948", "1949", "1950", "1951", "1952", "1953", "1954", "1955", "1958", "V1000", "V1001", "V1002", "V1003", "V1004", "V1005", "V1006", "V1007", "V1009", "V1011", "V1012", "V1020", "V1021", "V1022", "V1029", "V103", "V1040", "V1041", "V1042", "V1043", "V1044", "V1045", "V1046", "V1047", "V1048", "V1049", "V1050", "V1051", "V1052", "V1053", "V1059", "V1060", "V1061", "V1062", "V1063", "V1069", "V1071", "V1072", "V1079", "V1081", "V1082", "V1083", "V1084", "V1085", "V1086", "V1087", "V1088", "V1089", "V1090", "V1091"
Rheumatoid arthritis/collagen vascular diseases	"7010", "7011", "7012", "7013", "7014", "7015", "7018", "7019", "7100", "7101", "7102", "7103", "7104", "7105", "7108", "7109", "7140", "7141", "7142", "71430", "71431", "71432", "71433", "7144", "71481", "71489", "7149", "7200", "7201", "7202", "72081", "72089", "7209", "725"
Coagulopathy	"2860", "2861", "2862", "2863", "2864", "28652", "28653", "28659", "2866", "2867", "2869", "2871", "28730", "28731", "28732", "28733", "28739", "28741", "28749", "2875"
Obesity	"27800", "27801", "27802", "27803"
Weight loss	"260", "261", "262", "2630", "2631", "2632", "2638", "2639"
Fluid and electrolyte disorders	"2760", "2761", "2762", "2763", "2764", "27650", "27651", "27652", "27661",

	"27669", "2767", "2768", "2769"
Blood loss anemia	"2800"
Deficiency anemias	"2801", "2802", "2809", "2810", "2811", "2812", "2813", "2814", "2818", "2819", "2859"
Alcohol abuse	"2911", "2912", "2915", "29181", "29182", "29189", "2919", "30390", "30391", "30392", "30393", "30500", "30501", "30502", "30503", "V113"
Drug abuse	"2920", "29282", "29283", "29284", "29285", "29289", "2929", "30400", "30401", "30402", "30403", "30410", "30411", "30412", "30413", "30420", "30421", "30422", "30423", "30430", "30431", "30432", "30433", "30440", "30441", "30442", "30443", "30450", "30451", "30452", "30453", "30460", "30461", "30462", "30463", "30470", "30471", "30472", "30473", "30480", "30481", "30482", "30483", "30490", "30491", "30492", "30493", "30520", "30521", "30522", "30523", "30530", "30531", "30532", "30533", "30540", "30541", "30542", "30543", "30550", "30551", "30552", "30553", "30560", "30561", "30562", "30563", "30570", "30571", "30572", "30573", "30580", "30581", "30582", "30583", "30590", "30591", "30592", "30593"
Psychoses	"29500", "29501", "29502", "29503", "29504", "29505", "29510", "29511", "29512", "29513", "29514", "29515", "29520", "29521", "29522", "29523", "29524", "29525", "29530", "29531", "29532", "29533", "29534", "29535",

		"29540", "29541", "29542", "29543", "29544", "29545", "29550", "29551", "29552", "29553", "29554", "29555", "29560", "29561", "29562", "29563", "29564", "29565", "29570", "29571", "29572", "29573", "29574", "29575", "29580", "29581", "29582", "29583", "29584", "29585", "29590", "29591", "29592", "29593", "29594", "29595", "29600", "29601", "29602", "29603", "29604", "29605", "29606", "29610", "29611", "29612", "29613", "29614", "29615", "29616", "29620", "29621", "29622", "29623", "29624", "29625", "29626", "29630", "29631", "29632", "29633", "29634", "29635", "29636", "29640", "29641", "29642", "29643", "29644", "29645", "29646", "29650", "29651", "29652", "29653", "29654", "29655", "29656", "29660", "29661", "29662", "29663", "29664", "29665", "29666", "2967", "29680", "29681", "29682", "29689", "29690", "29699", "2970", "2971", "2972", "2973", "2978", "2979", "2980", "2981", "2982", "2983", "2984", "2988", "2989", "29910", "29911"
	Depression	"3004", "30112", "3090", "3091", "311"
	**In-hospital complication**	
	Hip dislocation	"83500", "83501", "83502", "83503", "83510", "83511", "83512", "83513", "99642"
	Periprosthetic fracture	"99644"
	Periprosthetic joint infection	"99666", "99667", "99669"

Mechanical complications	"99640", "99641", "99643", "99647", "99649"
Postprocedural hemorrhage	"99811"
Postprocedural hematoma	"99812"
Postprocedural seroma	"99813"
Wound dehiscence, superficial	"99831"
Wound dehiscence, deep	"99832"
Pressure ulcer	"70700", "70701", "70702", "70703", "70704", "70705", "70706", "70707", "70709", "70710", "70711", "70712", "70713", "70714", "70715", "70719", "70720", "70721", "70722", "70723", "70724", "70725", "7078", "7079"
Acute myocardial infarction	"41000", "41001", "41002", "41010", "41011", "41012", "41020", "41021", "41022", "41030", "41031", "41032", "41040", "41041", "41042", "41050", "41051", "41052", "41060", "41061", "41062", "41070", "41071", "41072", "41080", "41081", "41082", "41090", "41091", "41092"
Pneumonia	"4800", "4801", "4802", "4803", "4808", "4809", "481", "4820", "4821", "48230", "48231", "48232", "48239", "48240", "48241", "48242", "48249", "48281", "48282", "48283", "48284", "48289", "4829", "4830", "4831", "4838", "4841", "4843", "4845", "4846", "4847", "4848", "485", "486", "4870", "4871", "4878", "48801", "48802", "48809", "48811", "48812", "48819", "48881", "48882", "48889"

Respiratory failure	"5180", "51851", "51852", "51853", "51881", "51882", "51884"
Acute renal failure	"5845", "5846", "5847", "5848", "5849"
Cerebral infarction	"43400", "43401", "43410", "43411", "43490", "43491"
Peripheral nerve injury	"9560", "9561", "9562", "9563", "9564", "9568", "9569", "3550", "3551", "3552", "3553", "3554", "35571", "3557", "3558"
Inpatient death	N/A
Postoperative ileus	"5601"
Urinary tract infection	"5990"
**Patient medical history of:**	
Flexion contracture	"71840", "71845", "71955"
Valgus	"73631"
Varus	"73632"
Hip dislocation	"83500", "83501", "83502", "83503", "83510", "83511", "83512", "83513"
AVN	"73342"
JIA	"71430", "71431", "71432", "71433"
RA	"7140", "7141", "7144"
SLE	"7100"
ESRD	"5856"
Hardware removal	"V5401"

Spondyloarthropathy	“7200”, “7201”, “7202”, “72081”, “72089”, “7209”, “6960”, “0993”

**Spine condition**	**ICD-9 codes**
History of arthrodesis applied to all spinal fusions	“V454”
Spinal stenosis in cervical region	“7230”
Spinal stenosis in thoracic region	“72401”
Spinal stenosis in lumbar region without neurogenic claudication	“72402”
Spinal stenosis in lumbar region with neurogenic claudication	“72403”
Cervicalgia	“7231”
Radiculopathy	“7234”, “72290”, “72291”, “72292”, “72293”
Sciatica	“7243”
Lumbosacral neuritis NOS	“7244”
Cervical disc with myelopathy	“72271”
Thoracic disc with myelopathy	“72272”
Lumbar disc with myelopathy	“72273”
Cervical spondylosis with myelopathy	“7211”
Thoracic spondylosis with myelopathy	“72141”
Lumbar spondylosis with myelopathy	“72142”
